# Connections between EM2-containing terminals and GABA/μ-opioid receptor co-expressing neurons in the rat spinal trigeminal caudal nucleus

**DOI:** 10.3389/fncir.2014.00125

**Published:** 2014-10-24

**Authors:** Meng-Ying Li, Zhen-Yu Wu, Ya-Cheng Lu, Jun-Bin Yin, Jian Wang, Ting Zhang, Yu-Lin Dong, Feng Wang

**Affiliations:** ^1^Department of Nutrition and Food Hygiene, The Fourth Military Medical UniversityXi'an, China; ^2^Department of Anatomy, Histology and Embryology, K.K. Leung Brain Research Centre, The Fourth Military Medical UniversityXi'an, China

**Keywords:** endomorphin 2, γ-amino butyric acid, μ-opioid receptor, inhibitory interneuron, synapse, spinal trigeminal caudal nucleus

## Abstract

Endomorphin-2 (EM2) demonstrates a potent antinociceptive effect via the μ-opioid receptor (MOR). To provide morphological evidence for the pain control effect of EM2, the synaptic connections between EM2-immunoreactive (IR) axonal terminals and γ-amino butyric acid (GABA)/MOR co-expressing neurons in lamina II of the spinal trigeminal caudal nucleus (Vc) were investigated in the rat. Dense EM2-, MOR- and GABA-IR fibers and terminals were mainly observed in lamina II of the Vc. Within lamina II, GABA- and MOR-neuronal cell bodies were also encountered. The results of immunofluorescent histochemical triple-staining showed that approximately 14.2 or 18.9% of GABA-IR or MOR-IR neurons also showed MOR- or GABA-immunopositive staining in lamina II; approximately 45.2 and 36.1% of the GABA-IR and MOR-IR neurons, respectively, expressed FOS protein in their nuclei induced by injecting formalin into the left lower lip of the mouth. Most of the GABA/MOR, GABA/FOS, and MOR/FOS double-labeled neurons made close contacts with EM2-IR fibers and terminals. Immuno-electron microscopy confirmed that the EM2-IR terminals formed synapses with GABA-IR or MOR-IR dendritic processes and neuronal cell bodies in lamina II of the Vc. These results suggest that EM2 might participate in pain transmission and modulation by binding to MOR-IR and GABAergic inhibitory interneuron in lamina II of the Vc to exert inhibitory effect on the excitatory interneuron in lamina II and projection neurons in laminae I and III.

## Introduction

The superficial laminae (lamina I and lamina II) of the spinal trigeminal caudal nucleus (Vc), which is morphologically and functionally identical to the superficial laminae of the spinal dorsal horn and also named medullary dorsal horn, play a critical role in the transmission and modulation of oro-facial nociceptive information conveyed via primary afferents in the trigeminal nerve (Dubner and Bennett, 1983; Todd, 2010; Wu et al., 2010). Primary afferent fibers either contact projection neurons in lamina I directly or indirectly via interneurons in lamina II, through which they affect the activity of the interneurons and projection neurons (Dubner and Bennett, 1983; Wang et al., 2000, 2001; Todd, 2010; Wu et al., 2010). It has been reported that approximately 30% of lamina II interneurons show γ-amino butyric acid (GABA)-immunoreactivity in the Vc or spinal dorsal horn and GABAergic interneurons could be activated to regulate the nociceptive information transmission in the superficial laminae of the Vc (Todd et al., 1994; Wang et al., 2000, 2001; Yasaka et al., 2010). The results of our previous investigations have demonstrated that inflammatory nociceptive stimulation induced by subcutaneous injection of formalin into the lip could activate GABAergic neurons within the Vc exhibiting by expression functional marker FOS protein in the nuclei of the related neurons (Wang et al., 2000). Moreover, substance P (SP)-containing primary afferent terminals make asymmetric synapses with GABAergic interneurons in lamina II of the Vc (Wang et al., 2000) and GABAergic terminals originating from lamina II interneurons form symmetric synapses with projection neurons in lamina I and lamina III (Wang et al., 2001). These results suggest that inhibitory interneuronsplay an important role in regulating the excitatory neuronal activities in the Vc or spinal dorsal horn by releasing GABA (Dubner and Bennett, 1983; Todd et al., 1994; Wang et al., 2000, 2001; Todd, 2010; Wu et al., 2010; Yasaka et al., 2010).

Previous investigations have also demonstrated that endomorphins (EMs; including EM1 and EM2) are the specific endogenous ligands with the highest affinity to the μ-opioid receptor (MOR) (Zadina et al., 1997), and EM1- and EM2-immunopositive neuronal cell bodies are principally located in the hypothalamus and solitary tract nucleus in the central nerve system (CNS) (Martin-Schild et al., 1999). It has been reported that EM2 is more effective and potent in suppressing nociceptive information transmission than EM1, including pain relief in acute, inflammatory and neuropathic pain (Tseng, 2002). Administration or delivery of EM2, EM2 derivatives, EM2 analogues and EM2 gene recombination virus vectors have provided an analgesic effect in different pain models, which could be blocked by intrathecal delivery of the MOR antagonist, suggesting that EM2 might be used for the treatment of chronic pain (Przewlocka et al., 1999; Tseng, 2002; Fichna et al., 2013; Makuch et al., 2013; Varamini and Toth, 2013; Mizoguchi et al., 2014). EM2-immunoreactive (IR) fibers and terminals have been densely observed in the superficial laminae, particularly lamina II of the Vc (Martin-Schild et al., 1999). Most of the laminae are terminals of the primary afferent fibers originating from the EM2-containing neuronal cell bodies within the trigeminal ganglion (TG) (Zhu et al., 2011). Activities of the neurons in the superficial laminae are inhibited when EM2 was applied onto the spinal dorsal horn (Przewlocka et al., 1999; Tseng, 2002; Fichna et al., 2013; Makuch et al., 2013; Varamini and Toth, 2013; Mizoguchi et al., 2014). EM2-IR terminals have been found to make synaptic connections with the projection neurons in lamina I of the Vc (Aicher et al., 2003). Recently, it has been observed that EM2 and SP co-localized primary afferent terminals might regulate pain transmission in the spinal dorsal horn through co-releasing EM2 and SP to affect the activity of GABAergic interneurons in lamina II (Luo et al., 2014). There are MOR-IR neurons in lamina II of the Vc (Ding et al., 1996); however, morphological evidence to show that EM2 might modulate nociceptive information transmission through binding to MOR-expressing GABAergic interneuron in lamina II is still lacking. Based on these previous results, we proposed the hypothesis that EM2 might participate in oro-facial antinociception by releasing EM2 to act on GABA/MOR co-expression interneuron to regulate the activity of the projection neuron and interneuron, especially the excitatory interneuron, in lamina II of the Vc. To provide morphological evidence for the hypothesis, the connections between EM2-IR fibers and terminals and GABA/MOR co-localized neurons or nociceptive stimulation activated GABA- or MOR-IR neurons in lamina II of the Vc were investigated.

## Materials and methods

### Animals

Twenty one adult male Wistar rats (weighing 250–300 g) were used in the present study. The experimental procedures in this study were carried out in accordance with the National Institutes of Health Guide for the Care and Use of Laboratory Animals (NIH Publication No. 80-23) revised 1996 and IASP's guidelines for pain research in conscious animals (Zimmermann, 1983) and were approved by The Committee of Animal Use for Research and Education in The Fourth Military Medical University (Xi'an, China). All efforts were made to minimize animal suffering as well as the number of animals used.

The rats were divided into 3 groups (*n* = 6 for group 1 and 3, *n* = 9 for group 2). Group 1 was used for mapping the distribution pattern of EM2-, GABA- and MOR-IR neuronal structures, co-localization of GABA- and MOR-IR neurons in lamina II neurons and the close contacts between EM2-IR fibers and terminals and GABA/MOR co-localized neurons. Group 2 was used for subcutaneous injection of 5% formalin solution (100 μl) into the left lower lip of the mouth 2 h before the perfusion (Wang et al., 2000) and the connections between EM2-IR fibers and terminals and GABA/FOS or MOR/FOS double-immunopositive neurons in lamina II of the Vc. Group 3 was used for electron-microscopic immunohistochemical double-staining to observe the synaptic connections between EM2-IR terminals and GABA- or MOR-IR neuronal cell bodies and dendritic processes.

### Immunohistochemistry and immunofluorescent histochemistry

The rats from group 2 were anesthetized with diethyl ether inhalation and were then used for quick subcutaneous injection of 5% formalin solution (100 μl) into the left lower lip of the mouth (Wang et al., 2000). The same volume of saline was injected as control experiment. Two hours later, the rats from group 1 and group 2 were anesthetized with sodium pentobarbital (50 mg/kg) and then perfused transcardially with 100 ml of 0.9% saline, followed by 500 ml of 0.1 M phosphate buffer (PB, pH 7.4) containing 4% (w/v) paraformaldehyde and 0.2% (w/v) picric acid. The lower part of the brainstem, containing the medulla oblongata, were removed and postfixed in the same fixative for 4 h and then transferred to 30% sucrose in 0.1 M PB for cryoprotection at 4°C. The brainstem was transversely cut into 30 μm thick sections on a freezing microtome (Kryostat 1720; Leitz, Mannheim, Germany). The sections were divided into 6 serial sets and stored into 6 dishes containing 0.01 M phosphate-buffered saline (PBS, pH 7.4). Each dish contained a complete set of sections. Then, all sections were washed with 0.01 M PBS.

The immunohistochemical and immunofluorescent histochemical staining protocols used in the present study were the same as those in our previous study (Wang et al., 2000, 2001; Zhu et al., 2011; Kou et al., 2014; Luo et al., 2014); 4 sets of the sections from the first to fourth dishes from group 1 and one set of sections from the first dish from group 2 were incubated at room temperature sequentially with primary antibodies. All the antisera used in each group are shown in Table 1.

**Table 1:** **Antisera used in each group**.

**Group**	**Purposes**	**Primary antisera**	**Secondary antisera**	**Horseradish peroxides (HRP)- or fluorophore-conjugated *avidin***
Immuno-fluorescence	EM2	Rabbit anti-EM2 IgG(1:200, Abcam)	Biotinylated donkey anti-rabbit IgG (1:500, Millipore)	ABC Elite kit (1:100, Vector Labs)
	MOR	Guinea pig anti-MOR IgG(1:500, Millipore)	Biotinylated donkey anti-guinea pig IgG (1:500, Millipore)	ABC Elite kit (1:100, Vector Labs)
	GABA	Mouse anti-GABA IgG (1:200, Sigma)	Biotinylated donkey anti-mouse IgG (1:500, Millipore)	ABC Elite kit (1:100, Vector Labs)
	EM2/MOR/GABA	Rabbit anti-EM2IgG(1:200, Abcam)	Biotinylated donkey anti-rabbit IgG (1:500, Millipore)	Alexa 594-labeled avidin (1:1000, Molecular Probes)
		Guinea pig anti-MOR IgG (1:500, Millipore)	Alexa 647 donkey anti- guinea pig IgG (1:500, Molecular Probes)	
		Mouse anti-GABA IgG (1:200, Sigma)	Alexa 488 donkey anti-mouse IgG (1:500, Molecular Probes)	
	EM2/MOR/FOS	Rabbit anti-EM2 IgG(1:200, Abcam)	Biotinylated donkey anti-rabbit IgG (1:500, Millipore)	Alexa 594-labeled avidin (1:1000, Molecular Probes)
		Guinea pig anti-MOR IgG(1:500, Millipore)	Alexa 488 donkey anti- guinea pig IgG (1:500, Molecular Probes)	
		Goat anti-FOS IgG (1:200,Abcam)	Alexa 647 donkey anti-goat IgG (1:500, Molecular Probes)	
	EM2/GABA/FOS	Rabbit anti-EM2 IgG(1:200, Abcam)	Biotinylated donkey anti-rabbit IgG (1:500, Millipore)	Alexa 594-labeled avidin (1:1000, Molecular Probes)
		Mouse anti-GABA IgG (1:200,Sigma)	Alexa 488 donkey anti- mouse IgG (1:500, Molecular Probes)	
		Goat anti-FOS IgG (1:200, Abcam)	Alexa 647 donkey anti-goat IgG (1:500, Molecular Probes)	
Electron- microscopy	EM2/GABA	Rabbit anti-EM2 IgG (1:100, Abcam)	Biotinylated anti-rabbit IgG (1:200, Millpore)	ABC Elite kit (1:100, Vector Labs)
		Mouse anti-GABA IgG(1:100, Sigma)	Goat anti-mouse IgG antibody conjugated to 1.4 nm gold particles (1:100 Nanoprobes)	
	EM2/MOR	Rabbit anti-EM2 IgG(1:100, Abcam)	Biotinylated anti-rabbit IgG (1:200, Millpore)	ABC Elite kit (1:100, Vector Labs)
		guinea pig anti-MOR IgG(1:300, Millipore)	Goat anti-guinea pig IgG antibody conjugated to 1.4 nm gold particles (1:100, Nanoprobes)	

Briefly, the sections from group 1 were incubated with primary antibodies (1) rabbit anti-EM2 IgG (1:200, Abcam); (2) guinea pig anti-MOR IgG (1:500, Millipore); (3) mouse anti-GABA IgG (1:200, Sigma) and (4) the mixture of rabbit anti-EM2 IgG (1:200), guinea pig anti-MOR (1:500) and mouse anti-GABA (1:200) for 72 h in PBS containing 0.3% (v/v) Triton X-100, 0.25% (w/v) λ-carrageenan and 5% (v/v) donkey serum (PBS-XCD). Secondary antibodies included biotinylated donkey anti-rabbit IgG (1:500, Millipore), biotinylated donkey anti-guinea pig IgG (1:500, Millipore), biotinylated donkey anti-mouse IgG (1:500, Millipore) or a mixture of biotinylated donkey anti-rabbit IgG (1:500), Alexa Fluor 647-conjugated donkey anti-guinea pig IgG (1:500, Molecular Probes) and Alexa Fluor 488-conjugated donkey anti-mouse IgG (1:500, Molecular Probes) in PBS containing 0.3% (v/v) Triton X-100 for 4 h, followed by incubation with avidin-biotin-horseradish peroxides (HRP) complex or fluorophore-conjugated avidin, Alexa Fluor 594-conjugated avidin (1;1000, Molecular Probes), for 1 h. To demonstrate the HRP conjugation to the avidin-biotin complex, the sections from the first to third dishes from group 1 were treated with 0.05 M of Tris-HCl buffer (pH 7.6) containing 0.04% diaminobenzidinetetrahydrochloride (DAB) (Dojin, Kumamoto, Japan) and 0.003% H_2_O_2_ for 30 min.

The sections from group 2 were incubated with a mixture of primary antibodies as (1) rabbit anti-EM2 IgG (1:200), guinea pig anti-MOR IgG (1:500) and goat anti-FOS IgG (1:200, Abcam); (2) rabbit anti-EM2 IgG (1:200), mouse anti-GABA IgG (1:200) and goat anti-FOS IgG (1:200) in PBS-XCD for 72 h. Secondary antibodies were a mixture of (1) biotinylated donkey anti-rabbit IgG (1:500), Alexa Fluor 488-conjugated donkey anti-guinea pig IgG (1:500), and Alexa Fluor 647-conjugated donkey anti-goat IgG (1:500, Millipore) or (2) biotinylated donkey anti-rabbit IgG (1:500), Alexa Fluor 488-conjugated donkey anti-mouse IgG (1:500, Molecular Probes) and Alexa Fluor 647-conjugated donkey anti-goat IgG (1:500) in PBS containing 0.3% (v/v) Triton X-100 for 4 h, followed by incubation with fluorophore-conjugated avidin, Alexa Fluor 594-conjugated avidin (1;1000), for 1 h.

The specificities of the staining were tested on the sections in the fifth to seventh dishes from both group 1 and group 2 by omitting the specific primary antibodies. Neither immunopositive product nor immunoreactive labeling was found on these sections. The sections in the eighth dish from group 1 and group 2 were used for Nissl's staining.

Both incubated sections and reacted or stained sections were mounted onto gelatin-coated glass slides, air-dryed, dehydrated, and coverslipped. The sections were observed under a common light microscope (AHBT3; Olympus, Tokyo, Japan) for the DBA reacted sections or under a confocal laser-scanning microscope (Fluoview 1000, Olympus) for the immunofluorescent histochemically stained sections. Under the confocal laser-scanning microscope, all the sections were observed with appropriate laser beams and filter sets for Alexa 488 (excitation, 490 nm, emission, 525 nm), Alexa 594 (excitation, 590 nm, emission, 617 nm) or Alexa 647 (excitation, 650 nm, emission, ≥650 nm). Digital images were captured using Fluoview software (version 1.6; Olympus).

In each rat, the numbers of immuno-stained neurons in the Vc were counted through five randomly selected sections from each set and the ratios of different types of neurons were calculated. A careful review of the thickness of the selected sections determined that the immunolabeling had penetrated the entire thickness of the sections and only the neuronal cell bodies with obvious light emission were counted. The light from some positive neurons might be too weak to detect; therefore, the numbers of MOR-IR neurons and/or GABA-IR neurons in Tables 2–**4** should be regarded as representing the minimum of the real positive neurons in the sections.

**Table 2:** **Numbers of MOR-IR neuron, GABA-IR neuron and MOR/GABA co-expressing neuron**.

**Rat**	**MOR-IR neurons**	**GABA-IR neurons**	**MOR/GABA neurons**	**%^a^**	**%^b^**
R1-1	767	978	134	17.5	13.7
R1-3	806	1203	157	19.5	13.1
R1-5	738	894	145	19.6	16.2
Total	2311	3075	436	18.9	14.2

a The percentage of MOR/GABA double-labeled neurons to the total number of MOR-IR neurons;

b *The percentage of MOR/GABA double-labeled neurons to the total number of GABA-IR neurons*.

### Immuno-electron microscopy

For the electron microscopic study, rats in group 3 were deeply anesthetized and perfused transcardially with 100 ml of 0.9% saline followed by 500 ml of 0.1 M PB containing 4% paraformaldehyde, 0.05% glutaraldehyde and 0.2% picric acid. The lower brainstems were removed and postfixed in the same fixative without glutaraldehyde for 4 h at 4°C. The brainstem was transversely cut into 50 μm thick sections on a vibratome (Microslicer DTK-100; Dosaka, Kyoto, Japan). The sections were divided into two sets and collected into two dishes. To enhance the penetration of antibody, the sections were freeze-thawed in liquid nitrogen after cryoprotection. Details of these immuno-electron microscopy procedures were described in our previous studies (Wang et al., 2000; Li et al., 2002; Yasaka et al., 2010; Luo et al., 2014). Briefly, the sections were incubated with 0.05 M Tris-buffered saline (TBS; pH 7.4) containing 20% normal goat serum for 1 h to block non-specific immunoreactivity, and then collected in two dishes. The sections in each dish were incubated for 24 h at 4°C with a mixture of primary antibodies as (1) rabbit anti-EM2 IgG (1;100, Abcam) and guinea pig anti-MOR IgG (1:300, Millipore) (set 1) and (2) rabbit anti-EM2 IgG (1:100, Abcam) and mouse anti-GABA IgG (1:100, sigma) (set 2) (Table 1), respectively in 50 mM TBS containing 2% (v/v) normal goat serum (TBS-NGS). Then, a mixture of biotinylated donkey anti-rabbit IgG (1:200, Millipore) and goat anti-mouse IgG or goat anti-guinea pig IgG conjugated with 1.4 nm gold particles for GABA (1;100, Nanoprobes) or MOR (1:100, Nanoprobes) (Table 1) were incubated in TBS-NGS overnight at room temperature. Subsequently, the sections were processed with the following steps: (1) postfixation with 1% glutaraldehyde in 0.1 mol/L PB for 10 min; (2) silver enhancement with HQ Silver Kit (Nanoprobes); (3) incubation with ABC kit (Vector); (4) reaction with DAB and H_2_O_2_; (5) osmification; (6) counterstaining with uranylacetate. Ultrathin sections at 70 nm thickness were cut from lamina II of the Vc, mounted on single-slot grids, and examined with an electron microscope (JEM1440, Tokyo, Japan).

## Results

**Figure 1:**
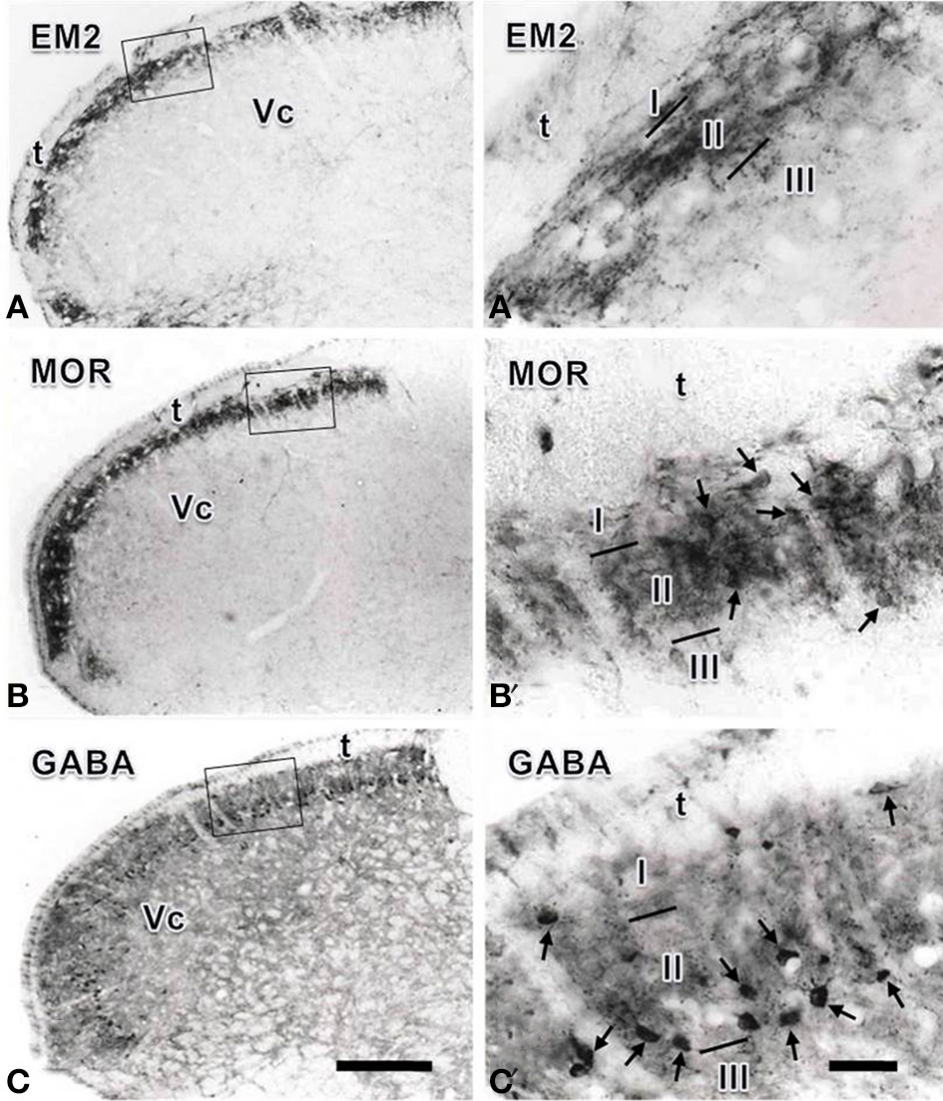
**Immunohistochemical staining showing the distributions of endomorphin-2 (EM2, A), μ-opioid receptor (MOR, B) and GABA (C) immunoreactive (IR) neuronal cell bodies and/or fibers and terminals in the spinal trigeminal caudal nucleus (Vc)**. The rectangle areas in **(A–C)** are enlarged in **(A′–C′)**, respectively. Arrows in **(B′,C′)** point to MOR- and GABA-IR neuronal cell bodies, respectively. I, lamina I; II, lamina II; III, lamina III; t, spinal trigeminal tract. Scale bars = 320 μm (in **C** and also for **A** and **B**) and 60 μm (in **C′** and also for **A′** and**B′**).

In the Vc, dense EM2-, MOR- and GABA- IR short-filate-like fibers and terminals punctiform in shape were found to be concentrated in the superficial laminae (lamina I and lamina II), especially in lamina II of the Vc (Figure 1). In lamina III of the Vc, only sparsely distributed EM2-, MOR- and GABA-IR fibers and terminals were found (Figure 1). A few MOR- and GABA-IR neuronal cell bodies were also located in laminae I- III, especially in lamina II (Figures 1B′,C′), but none of EM2-IR neuronal cell bodies were seen in the Vc (Figure 1A′). In lamina I and lamina III only a small number of sparsely distributed MOR- and GABA-IR neurons were encountered. MOR- and GABA-IR neuronal cell bodies were spherical, ovoid, fusiform or triangular in shape, and the diameters of cell bodies of these neurons ranged from 15 to 30 μm (Figures 1B′,C′).

**Figure 2:**
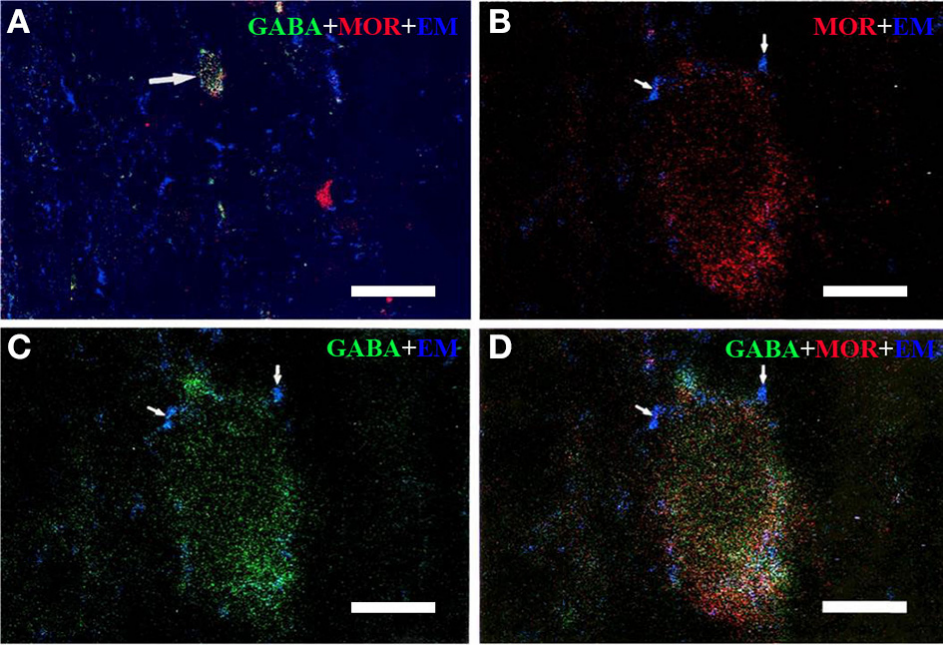
**Immunofluorescent histochemical triple-staining showing the connections between EM2-immunoreactive (IR) terminals (A–D, blue) and neuronal cell bodies exhibiting both MOR-IR (B, red) and GABA-IR (C, green) positive staining in lamina II of the Vc**. The merged image in **(D)** reveals close contacts between EM2-IR axon terminals and MOR/GABA co-localized neuronal cell bodies (yellow). The arrow in **(A)** showed a MOR/GABA co-localized neuron connecting with EM2-IR axonal terminals. The arrows in **(B–D)** showed connections between EM2-IR axonal terminals and MOR or GABA-IR neuronal cell body. Scale bars = 45 μm **(A)**, 6 μm (in **B–D**).

**Figure 3:**
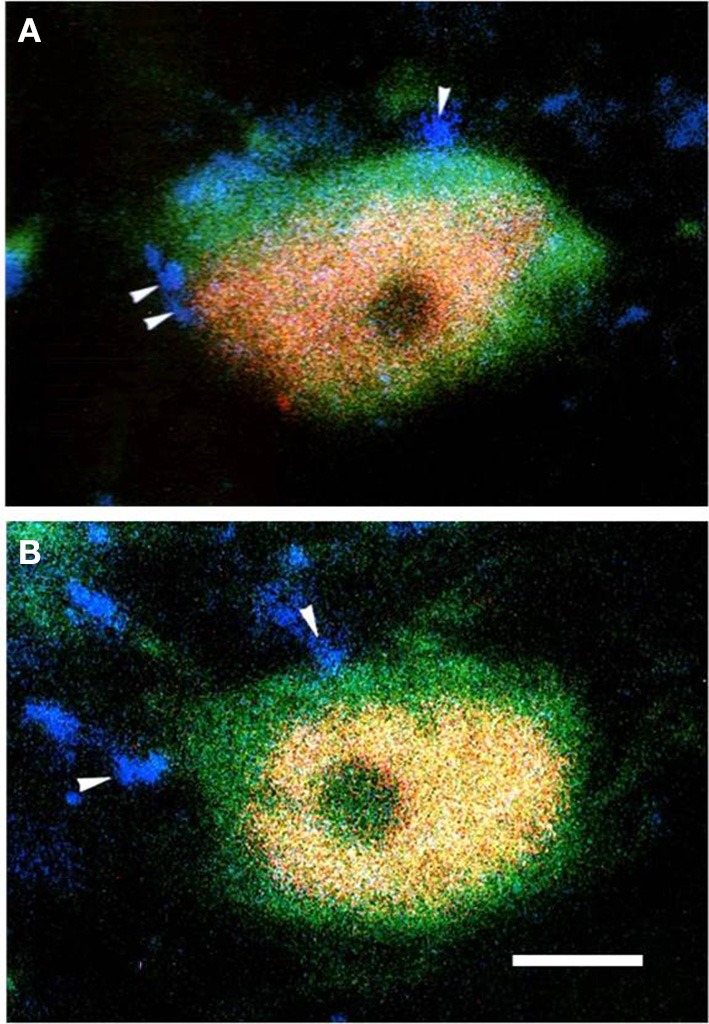
**The fluorescence photomicrographic images of triple-labeling showing the connections between EM2-IR terminals and GABA/FOS or MOR/FOS double-labeled neurons in lamina II of the Vc ipsilateral to the formalin injecting into the lower lip of the mouth**. Some of the EM2-IR terminals (**A**,**B**; blue, arrowheads) were in close contacts with GABA (green) and FOS (red) double-labeled neurons **(A)** and with MOR (green) and FOS (red) double-labeled neurons **(B)**. Scale bar = 8 μm.

Triple-immunofluorescent histochemical staining revealed that a small number of neurons contained both MOR- and GABA-immunopositive reaction products. Quantitative analysis showed that approximately 18.9% of MOR-IR neuronal cell bodies contained both MOR- and GABA-immunoreactivity and approximately 14.2% of GABA-IR neuronal cell bodies were immunoreactive for both MOR and GABA (Table 2). MOR/GABA co-localized neuronal cell bodies share the same morphological features of the MOR-IR or GABA-IR neurons. Then, we observed that some of these MOR- and GABA-IR co-localized neuronal cell bodies and their processes were in close contacted with EM2-IR fibers and terminals in the Vc (Figure 2, Supplementary Figure 1). In the formalin lip injected rats, triple-immunofluorescent histochemical staining also revealed that approximately 58% of the MOR-IR neurons or 79.8% of the GABA-IR neurons expressed FOS protein principally in lamina II of the Vc (Table 3); 27.6% and 47.7% of the FOS-IR and MOR-IR neurons co-expressed MOR and FOS (Table 3); and 36.1 and 45.2% of the FOS-IR and GABA-IR neurons co-localized GABA and FOS (Table 3). EM2-IR fibers and terminals were also observed to be in close contacts with the MOR/FOS or GABA/FOS double-labeled neurons in the Vc (Figure 3). Only a few FOS immunoreactivities were observed in the Vc in saline injected group (Supplementary Figure 2).

**Table 3:** **Numbers of MOR-IR neuron, GABA-IR neuron and FOS/MOR or FOS/GABA co-expressing neuron**.

**Rat**	**FOS-IR neurons**	**MOR-IR neurons**	**GABA-IR neurons**	**MOR/FOS neurons**	**GABA/FOS neurons**	**%^a^**	**%^b^**	**%^c^**	**%^d^**	**%^e^**	**%^f^**
R2-1	1279	723	915	323	407	56.5	71.5	25.3	44.7	31.8	44.5
R2-3	1206	694	1045	381	476	57.5	86.7	31.6	54.9	39.5	45.6
R2-5	1178	706	962	308	438	59.9	81.7	26.1	43.6	37.2	45.5
Total	3663	2123	2922	1012	1321	58.0	79.8	27.6	47.7	36.1	45.2

a The percentage of MOR-IR neurons to the total number of FOS-IR neurons;

b The percentage of GABA-IR neurons to the total number of FOS-IR neurons;

c The percentage of MOR/FOS co-expressing neurons to the total number of FOS-IR neurons;

d The percentage of MOR/FOS co-expressing neurons to the total number of MOR-IR neurons;

e The percentage of GABA/FOS co-expressing neurons to the total number of FOS-IR neurons;

f *The percentage of GABA/FOS co-expressing neurons to the total number of GABA-IR neurons*.

**Figure 4:**
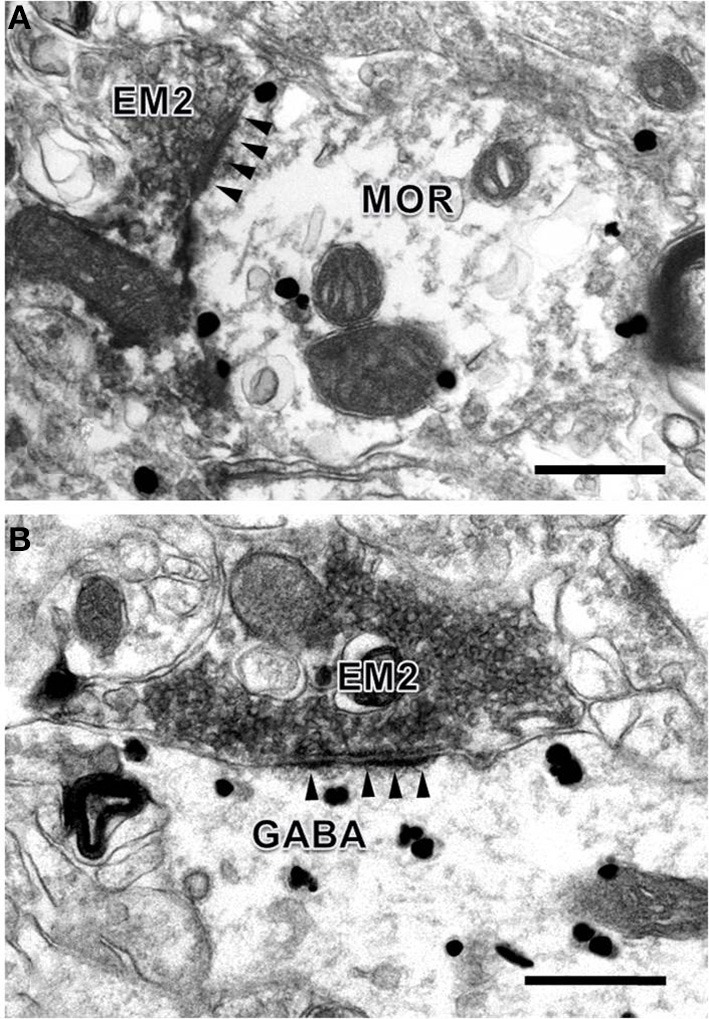
**Synaptic connections between EM2-IR axon terminals and MOR-IR and GABA-IR structures in lamina II of the Vc**. EM2-IR pre-synaptic axon terminals **(A**,**B**; EM2; filled with DAB reaction products) made asymmetric synapses with a post-synaptic MOR-IR **(A)** or GABA-IR **(B)** dendritic process, both of which were labeled with immune-gold particles. Arrowheads indicate post-synaptic membranes. Scale bars = 300 nm **(A)**, 400 nm (in **B**).

To provide convincing morphological evidence for these connections observed under light microscopy, electron microscopy was performed subsequently to demonstrate the synaptic connections between the EM2-IR fibers and terminals and MOR-IR or GABA-IR neurons. Under the electron microscope, EM2-IR axonal terminals, usually filled with synaptic vesicles, were characterized by the presence of electron dense DAB reaction products adhering to the outer surface of cell organelles such as mitochondria and synaptic vesicles and the inner surface of the plasma membrane (Figure 4). MOR-immunoreactivity was determined by the presence of the immunogold-silver grains distributed in the cytoplasm of the neuronal cell bodies, dendrites, and axonal fibers and terminals of the MOR-IR neurons. These gold particles trended to be localized beneath the membrane of the neuronal cell bodies, dendrites, and axons (Figure 4A). GABA-IR staining was exhibited by the presence of the immunogold-silver particles localized homogenously in both cytoplasm of the neuronal cell bodies and their processes (Figure 4B) of the GABA-IR neurons. For the sections from the first dish, a total of 153 EM2-IR axon terminals were found to mainly make asymmetric axo-dendritic and axo-somatic synapses with MOR-IR dendritic processes (Figure 4A) and MOR-IR neuronal cell bodies (Table 4). For the sections from the second dish, 181 EM2-IR axon terminals were also observed to form asymmetric axo-dendritic and axo-somatic synapses with GABA-IR dendritic processes (Figure 4B) and GABA-IR somatic profiles (Table 4).

**Table 4:** **Synaptic types between EM2-IR terminals and MOR-IR or GABA-IR sotamic profiles and dendritic processes**.

	**Symmetric synapses**	**Asymmetric synapses**	**Symmetric synapses**	**Asymmetric synapses**	**Symmetric synapses**	**Asymmetric synapses**
	**MOR-IR somatic profiles**		**MOR-IR dendritic processes**		**Total**	
EM2-IR terminal	1 (0.7%)	12 (7.9%)	2 (1.3%)	138 (90.1%)	3 (2.0%)	150 (98.0%)
	**GABA-IR somatic profiles**		**GABA-IR dendritic processes**		**Total**	
EM2-IR terminal	0 (0%)	15 (8.3%)	1 (0.5%)	165 (91.2%)	1 (0.5%)	181 (99.5%)

## Discussion

The superficial laminae (lamina I and lamina II) of the spinal trigeminal caudal nucleus (Vc, also named medullary dorsal horn) are critical for oro-facial nociceptive information transmission and regulation (Dubner and Bennett, 1983). There are many inhibitory interneurons located in lamina II (substantia gelatinosa) of the Vc and they are fundamental for nociceptive modulation (Dubner and Bennett, 1983; Todd et al., 1994; Wang et al., 2000, 2001; Todd, 2010; Wu et al., 2010; Yasaka et al., 2010). Therefore, in the present study, morphological methods were used to investigate the connections between the primary afferent EM2-containing fibers and terminals and MOR-expressing and GABA-containing inhibitory interneurons related to oro-facial nociceptive information transmission and/or modulation in the superficial laminae of the Vc. Our previous results have shown that SP released from the primary afferent fibers may activate GABAergic inhibitory neurons in lamina II (Wang et al., 2000). GABAergic inhibitory neurons may inhibit the activity of ascending projection neurons in lamina I of the Vc (Wang et al., 2001). These results suggest that GABAergic interneurons might play an important role in regulating the transmission of oro-facial nociceptive information to the medullary dorsal horn.

Here, we found that EM2-IR fibers and terminals make close contacts with the MOR/GABA co-localized neurons and GABAergic or MOR-IR neurons in the superficial laminae of the Vc, especially in lamina II were activated by peripheral noxious stimulation. Additionally, EM2-IR fibers and terminals were also observed to form asymmetric synapses with GABAergic neurons or MOR-IR neurons in the present study.

It has been demonstrated that EM2, a MOR agonist, is released from the presynaptic site, i.e., the primary afferent terminals coming from the primary sensory neurons in trigeminal ganglion (TG). The released EM2 might bind to the MOR autoreceptor located on the primary afferent terminals, to further inhibit SP release attenuating the sensation of pain and alleviating neuropathic pain (Li et al., 1998; Greenwell et al., 2007; Luo et al., 2014). Therefore, the interaction between EM2 and SP may affect the modulation of nociceptive information transmitted from the peripheral to the CNS. Previous studies focused on the co-localization of EM2-containing primary afferents and pre-synaptic pain control mechanism of the EM2 (Li et al., 1998; Greenwell et al., 2007; Luo et al., 2014), but less on the post-synaptic mechanism, especially for the EM2-IR targeted interneurons in lamina II containing both MOR and inhibitory transmitters, such as GABA. GABA is believed to be involved in both pre-synaptic and post-synaptic inhibition in the superficial laminae of the spinal and medullary dorsal horns (Heinke et al., 2004). GABAergic inhibitory interneurons in the spinal and medullary dorsal horns can be activated by subcutaneous injection of formalin into the hind paw or lip of mouth in the rat, and these GABAergic neurons can also express FOS after noxious stimulation (Todd et al., 1994; Wang et al., 2000). The present results provide a morphological evidence for our hypothesized connections between direct connections between EM2-IR fibers and terminals and MOR/GABA co-localized neurons mainly in lamina II of the Vc. However, because of the limitation of methods, it is difficult to make triple-labeling for EM2, GABA, and MOR simultaneously on the same section under electron microscope. It is well worth observation of synaptic connection between EM2-IR axonal terminals and the GABA/MOR double labeling neuronal profiles after new pre-embedding method coming.

As mentioned above, these results suggest that there are potential modulatory interactions between EM2-IR primary afferent fibers and terminals and lamina II interneurons through both pre-synaptic and post-synaptic mechanisms, especially through MOR-expressing GABAergic inhibitory interneurons, in nociceptive information transmission and modulation in both spinal and medullar dorsal horns. The present results also indicate that connections between EM2-IR fibers and terminals and MOR/GABA co-localized neurons in the superficial laminae of the Vc might be very important for the regulation of the peripheral oro-faical nociceptive transmission.

## Author contributions

Study concept and design: Yu-Lin Dong and Feng Wang. Light microscopic study: Meng-Ying Li and Zhen-Yu Wu. Electron microscopic study: Ya-Cheng Lu and Yu-Lin Dong. Data analysis: Jun-Bin Yin and Jian Wang. Technical support: Ting Zhang. Manuscript writing: Meng-Ying Li, Zhen-Yu Wu, Yu-Lin Dong, and Feng Wang.

### Conflict of interest statement

The authors declare that the research was conducted in the absence of any commercial or financial relationships that could be construed as a potential conflict of interest.
