# Supplementary material for: Connections between EM2-containing terminals and GABA/μ-opioid receptor co-expressing neurons in the rat spinal trigeminal caudal nucleus
Source: Front Neural Circuits. 2014 Oct 24;8:125. doi: 10.3389/fncir.2014.00125 (PMC4208411; doi:10.3389/fncir.2014.00125)
Supplement: Supplementary file 1 [file Presentation1.PDF]

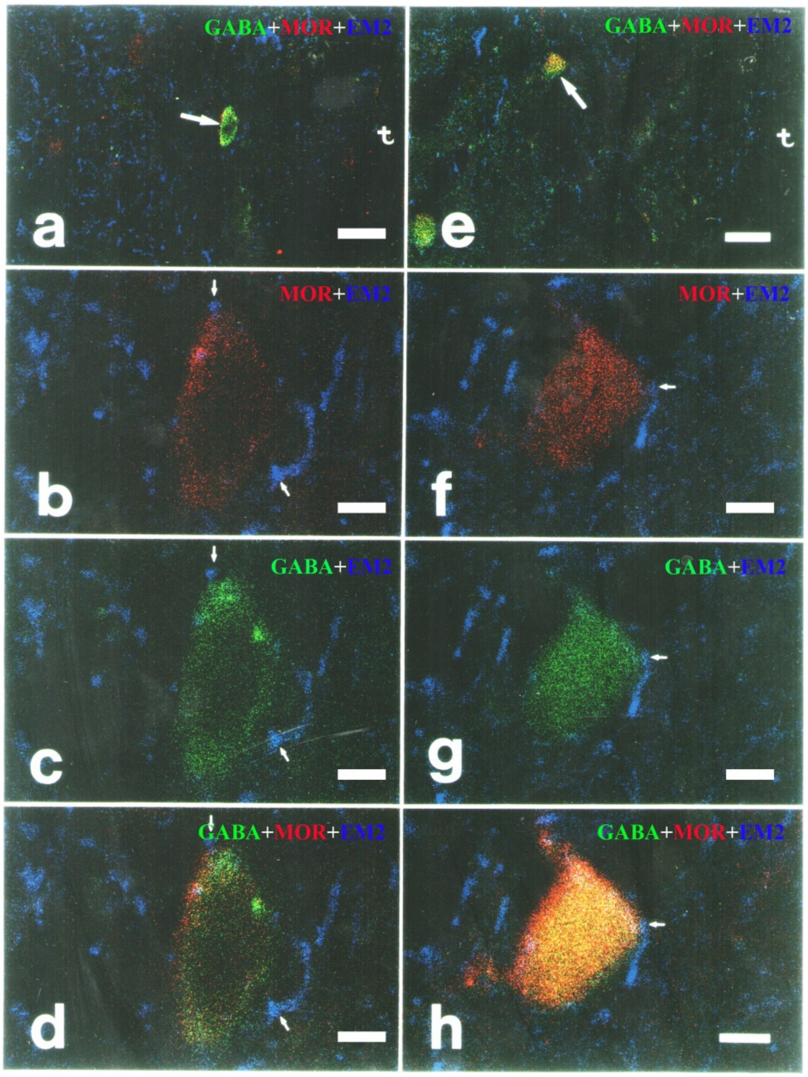

Supplementary Fig.1 Immunofluorescent histochemical triple-staining showing the connections between EM2-immunoreactive (IR) terminals (**a-h**, blue) and neuronal cell bodies exhibiting both MOR-IR (**b and f**, red) and GABA-IR (**c and g**, green) positive staining in lamina II of the Vc. The merged image in **d and h** reveals close contacts between EM2-IR axon terminals and MOR/GABA co-localized neuronal cell bodies (yellow). The arrows in Fig.a and e showed MOR/GABA co-localized neuronal cell bodies connecting with EM2-IR axonal terminals. The arrows in Fig.b-d and f-g showed connections between EM2-IR axonal terminals and MOR or GABA-IR neuronal cell bodies. t: spinal trigeminal tract. Scale bars = 45 μm (**a and e**), 6 μm (in **b-d and f-h**).
