# Supplementary material for: Connections between EM2-containing terminals and GABA/μ-opioid receptor co-expressing neurons in the rat spinal trigeminal caudal nucleus
Source: Front Neural Circuits. 2014 Oct 24;8:125. doi: 10.3389/fncir.2014.00125 (PMC4208411; doi:10.3389/fncir.2014.00125)
Supplement: Supplementary file 2 [file Presentation2.PDF]

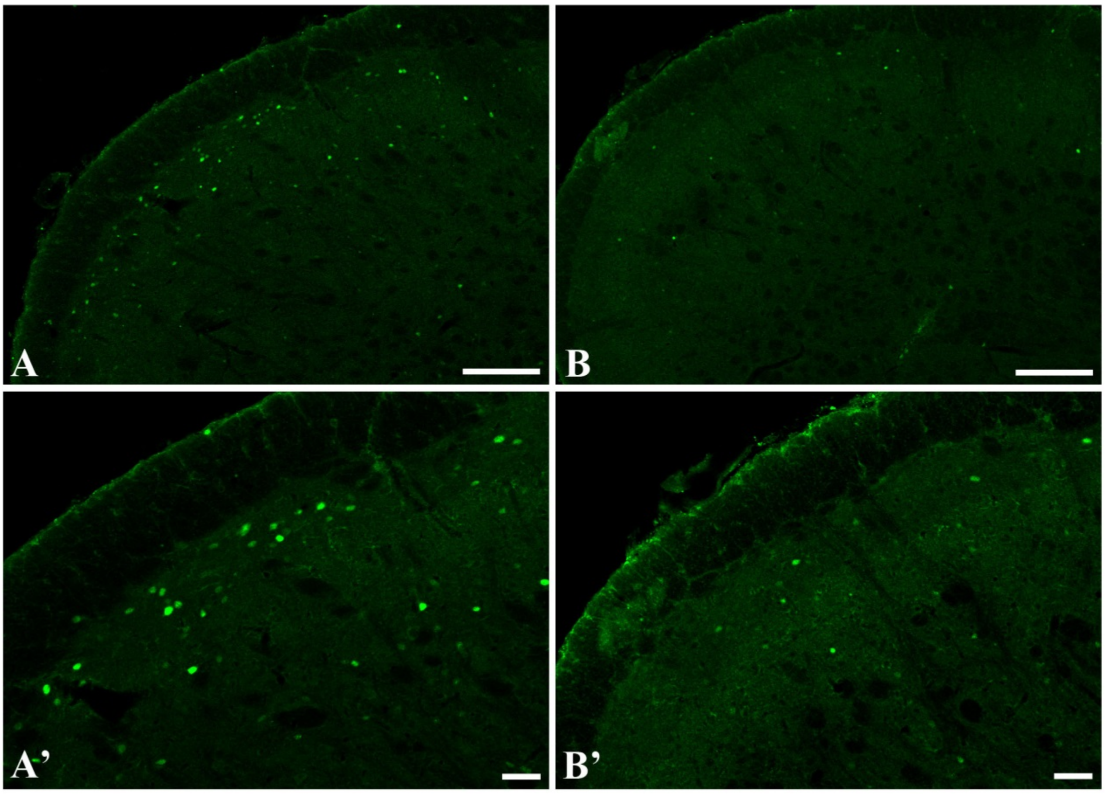

Supplementary Fig.2 Immunofluorescent images showing Fos expression induced by formalin-injection. A and A’: formalin injected animal; B and B’: saline injected animal. A’ and B’ showed the high magnification of figure A and B. Scale bars = 200 μm (**A and B**), 50 μm (A’ and B’)
